# Preliminary Expression Analysis of the *OSCA* Gene Family in Maize and Their Involvement in Temperature Stress

**DOI:** 10.3390/ijms232113658

**Published:** 2022-11-07

**Authors:** Yuanyang Li, Yubin Zhang, Bin Li, Liyuan Hou, Jianing Yu, Chengguo Jia, Zhe Wang, Siqi Chen, Mingzhe Zhang, Jianchun Qin, Ning Cao, Jinhu Cui, Wuliang Shi

**Affiliations:** Center for Emerging Agricultural Education & Advanced Interdisciplinary Science, College of Plant Science, Jilin University, Changchun 130062, China

**Keywords:** calcium signaling, OSCA, DUF221 domain, abiotic stress, maize (*Zea mays* L.)

## Abstract

Hyperosmolality-gated calcium-permeable channels (OSCA) are characterized as an osmosensor in plants; they are able to recognize and respond to exogenous and endogenous osmotic changes, and play a vital role in plant growth and adaptability to environmental stress. To explore the potential biological functions of OSCAs in maize, we performed a bioinformatics and expression analysis of the *ZmOSCA* gene family. Using bioinformatics methods, we identified twelve *OSCA* genes from the genome database of maize. According to their sequence composition and phylogenetic relationship, the maize *OSCA* family was classified into four groups (Ⅰ, Ⅱ, Ⅲ, and Ⅳ). Multiple sequence alignment analysis revealed a conserved DUF221 domain in these members. We modeled the calcium binding sites of four OSCA families using the autodocking technique. The expression profiles of *ZmOSCA* genes were analyzed in different tissues and under diverse abiotic stresses such as drought, salt, high temperature, and chilling using quantitative real-time PCR (qRT-PCR). We found that the expression of twelve *ZmOSCA* genes is variant in different tissues of maize. Furthermore, abiotic stresses such as drought, salt, high temperature, and chilling differentially induced the expression of twelve *ZmOSCA* genes. We chose *Zm*OSCA2.2 and *Zm*OSCA2.3, which responded most strongly to temperature stress, for prediction of protein interactions. We modeled the calcium binding sites of four OSCA families using autodocking tools, obtaining a number of new results. These results are helpful in understanding the function of the plant *OSCA* gene family for study of the molecular mechanism of plant osmotic stress and response, as well as exploration of the interaction between osmotic stress, high-temperature stress, and low-temperature stress signal transduction mechanisms. As such, they can provide a theoretical basis for crop breeding.

## 1. Introduction

As a major food crop, maize (*Zea mays* L.) plays a vital role in solving world hunger problems. However, the production process of maize is highly dependent on suitable environmental factors [1]. With the reduction in the availability and quality of arable land and water resources, as well as frequent extreme weather [2], many different types of abiotic stresses have been highlighted, such as salinity, drought, heat, and chilling [3]. These major abiotic environmental stresses seriously affect crop development and constrain agronomic yields worldwide. Supposedly, abiotic stresses may be responsible for a yield reduction of over 50% in major crop plants globally [4]. Studies have shown that the decline in maize yield is very significant under conditions of undesirable stress. In the last 30 years, maize yield losses due to heat stress were 1.13% per decade in China [5]. Based on reliable data, future high temperatures are predicted to increase the decline in corn production from 7% to 31% [6]. High temperatures are currently a major constraint on corn yields in Northeast China [7]. Cold stress limits food production, and is detrimental to normal plant growth and development [8]. Low temperatures disrupt photosynthetic metabolism, and may produce stress products such as reactive oxygen species, leading to a reduction in nutrients as well as inducing harmful structural changes in cells [9]. Maize growth in the field is influenced by various environmental factors, and it is particularly relevant to elucidate the signaling patterns associated with resistance to stress.

Temperature, salt, and drought stresses are major environmental factors that affect growth and development of plants in world, restrict plant productivity, and threaten human food security. How plants sense these stress signals and adapt to adverse environments are fundamental biological questions [10]. Under osmotic stress, an increase in intracellular calcium levels is achieved through transport pathways such as calcium channels [11]. Plants are subject to adversity stress, which triggers calcium oscillations in plants [12]; at the physiological, cellular, and molecular levels, this takes the form of inducing the expression of many stress-related genes, regulating plant tolerance to stress, and generating specific calcium signals [13]. It has been reported that calcium-associated proteins are required for several environmental stress responses [14]. Ion channels are proteins located in the cell membrane that aid in ion transport and play an important role in maintaining cellular homeostasis [15]. Hyperosmolality-gated calcium-permeable channels (OSCA), as one of the more recently discovered calcium channels, play an essential role in calcium signaling. Following the discovery and identification of *OSCA*1.1 in *Arabidopsis thaliana* [16], the *OSCA* gene family has been increasingly valued by scientists worldwide. Yuan et al. [16] established that OSCA1 protein, localized in the plant cell membrane, is expressed in both leaves and roots of *Arabidopsis*. Currently, *ZmOSCA* is associated with osmolarity-related stresses [17] such as drought and ABA, which is consistent with studies on other species. Over time, researchers have discovered that *OSCA* is widely distributed in eukaryotic cells, whether plants or animals [18]. It has been discovered that *OSCA* carries a major conserved protein, DUF221, which contains six predicted TM helices [19]. The TM helices in the structures of such proteins may have disappeared during evolution, as *OsOSCA2.2* lacks three TM regions [20]. The same study found that OSCA proteins may have effects on other traits. *TaOSCA1.4*, which belongs to the same family as *AtOSCA1.8* and *OsOSCA1.4* in the evolutionary tree analysis, is related to the grain number per ear [21]. The number of grains on the ear is one of the most important traits for maize yield. In addition, osmotic pressure affects the water balance in the plant, which in turn determines the crop yield. *OSCA* is a confirmed gene family associated with osmolality, and we found that it may have a key role in coping with the effects of temperature as well. This is an important means of improving the knowledge of abiotic stress resistance in maize. More importantly, determining the stress tolerance mechanism of maize can provide a theoretical basis for breeding drought-resistant varieties and improving cultivation techniques.

In the present study, we performed a bioinformatics analysis of the entire maize genome and identified twelve *OSCA* genes. These twelve maize *OSCAs* were grouped based on their phylogenetic relationships and their expression profiles in various organs and under different abiotic stresses. We found that *OSCAs* play a role in osmolality, and may be important in temperature stress and related signaling pathways as well. Furthermore, we predicted the calcium binding sites in proteins and carried out a protein interaction analysis, verifying that our conjecture is possible. These results can be used for functional validation studies of *ZmOSCA* and increase our understanding of the roles of plant *OSCAs*.

## 2. Results

### 2.1. Phylogenetic Relationship and Gene Structure of Maize OSCAs

**Figure 1:**
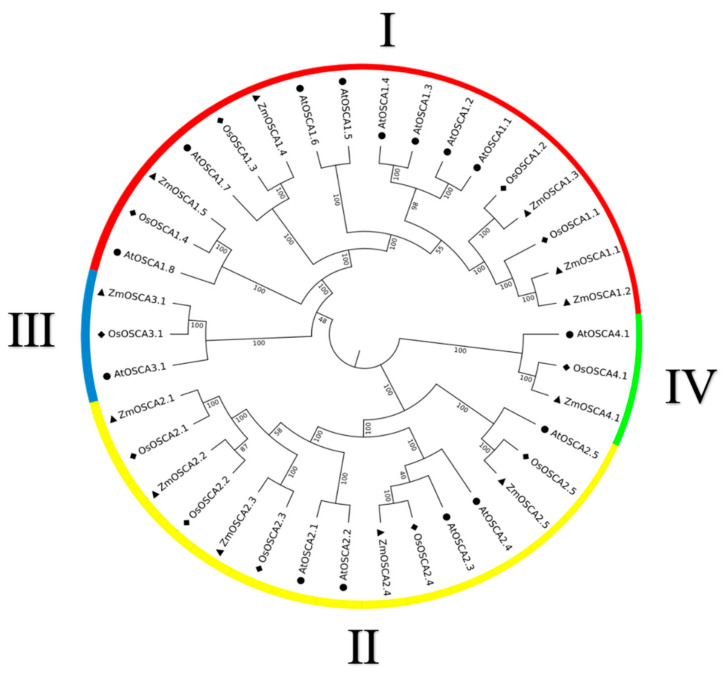
Phylogenetic analysis of OSCA proteins. *Arabidopsis*, rice, and maize are denoted by circle, diamond, and triangle respectively, and the tree is divided into four groups.

**Figure 2:**
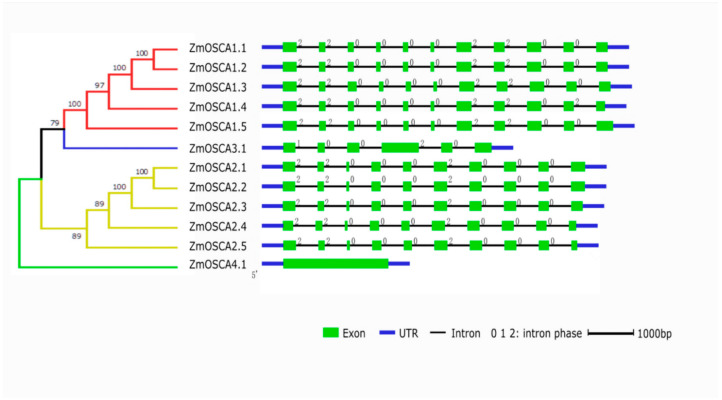
Exon–intron gene structures of *ZmOSCAs*. The horizontal black lines, green boxes, thick blue lines, and numbers in the top right corner of the green boxes indicate the positions of introns, the positions of exons, the positions of UTRs (untranslated regions), and the intron phase, respectively.

We used the CDS (coding sequence of amino acids in protein) of 38 *OSCAs* from rice, *Arabidopsis*, and maize to construct a phylogenetic tree (Figure 1) to determine the evolutionary relationships of *OSCA* in maize, and collated the information related to the *ZmOSCA* gene in the maize genome database (http://ensembl.gramene.org/, revised 18 January 2022) (Table 1). We found that members of the *OSCA* family were separated into four distinct clades, designated I, II, III, and IV (Figure 2). Most *OSCA* family members in *Arabidopsis*, rice, and maize were mainly distributed in groups I and Ⅱ as compared with groups III and IV. By further analyzing the results, we found that the *OSCA* genes in maize and rice were more closely related. We grouped all the *OSCAs* genes as shown in Table 2.

Then, we used Gene Structure Display Server (http://gsds.cbi.pku.edu.cn/index.php, revised 30 January 2022) to generate the intron and exon structure information maps of each gene in the *ZmOSCA* family (Figure 2). It was recognized that the gene structures of *OSCA* genes belonging to the same subfamily were often very similar, with the number and size of introns and exons being nearly identical. Except for *ZmOSCA3.1*, which contained six exons and for which the fourth exon was significantly longer, all members of the ZmOSCA I subfamily contained eleven exons and ten introns; however, the lengths of exons 1, 2, 5, and 6 of *ZmOSCA3.1* are noteworthy. The lengths of exons 1, 2, 5, and 6 of *ZmOSCA3.1* are the same as the lengths of the exons at both ends of the other members. All OSCA II subfamily members contain ten exons and nine introns. *ZmOSCA4.1* is more distantly related to the other members, and may be more primitive and conserved.

### 2.2. Analysis of Structural Domains of the ZmOSCA Family

**Figure 3:**
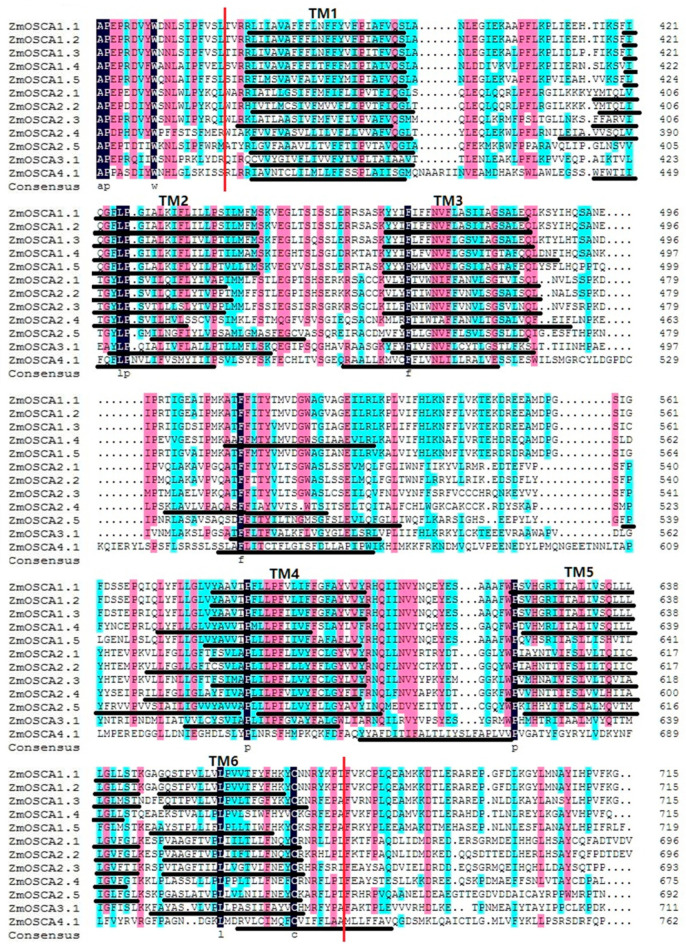
Multiple sequence alignment and transmembrane region of the DUF221 conserved region in ZmOSCAs. The region between two vertical red lines represents the DUF221 conserved region. Identical (100%), conserved (75–99%), and blocks (50–74%) of similar amino acid residues are shaded in dark navy, pink, and cyan, respectively. The transmembrane regions are marked by black lines and called TM1-TM6.

DUF221 is a characterized structural domain of the *OSCA* gene family [16]. Multiple sequence alignments of *OSCAs* were performed using DNAMAN software (http://www.lynnon.com/dnaman.html, revised 20 February 2022), and the transmembrane (TM) region of the DUF221 conserved region was predicted using TMHMM. We found that different OSCA members contain different TMs; most of the OSCA have six TMs with two exceptions. ZmOSCA4.1 had fewer TM4, and ZmOSCA1.5 had more TM5 disappear than their orthologues, which suggested that a deletion event occurred in the genomes of *ZmOSCA4.1* and *ZmOSCA1.5* (Figure 3). TMs from the same family are similar, while there are large differences between families.

### 2.3. Predicted Secondary Structure Analysis of ZmOSCA Gene Family Proteins

**Figure 4:**
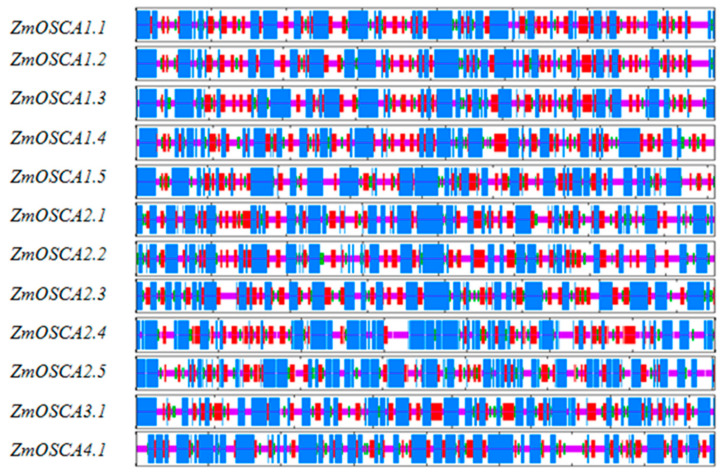
Predicted secondary structure of maize OSCA proteins. The blue, red, green, and yellow stripes represent the α helices, the β bridges, the β turns, and the random coils, respectively.

The secondary structure of each member of the ZmOSCA protein family was predicted by SOPMA based on the Protein Data Bank, and the results were counted. The results showed that the twelve OSCA protein family members of maize mainly contained four structures, Alpha helix (Hh), Extended strand (Ee), Beta turn (Tt), and Random coil (Cc), among which Alpha helix (Hh) accounted for the largest number and proportion of the secondary structures (Figure 4: Table 3). The protein secondary structures of *Zm*OSCA1.1, *Zm*OSCA1.2, and *Zm*OSCA1.3 are identical to each other. *Zm*OSCA2.1, *Zm*OSCA2.2, and *Zm*OSCA2.3 are very close to each other as well. In addition, we discovered a large difference in the secondary structure of proteins from different families.

### 2.4. Characterization of the ZmOSCA Gene Family for Tissue-Based Expression

**Figure 5:**
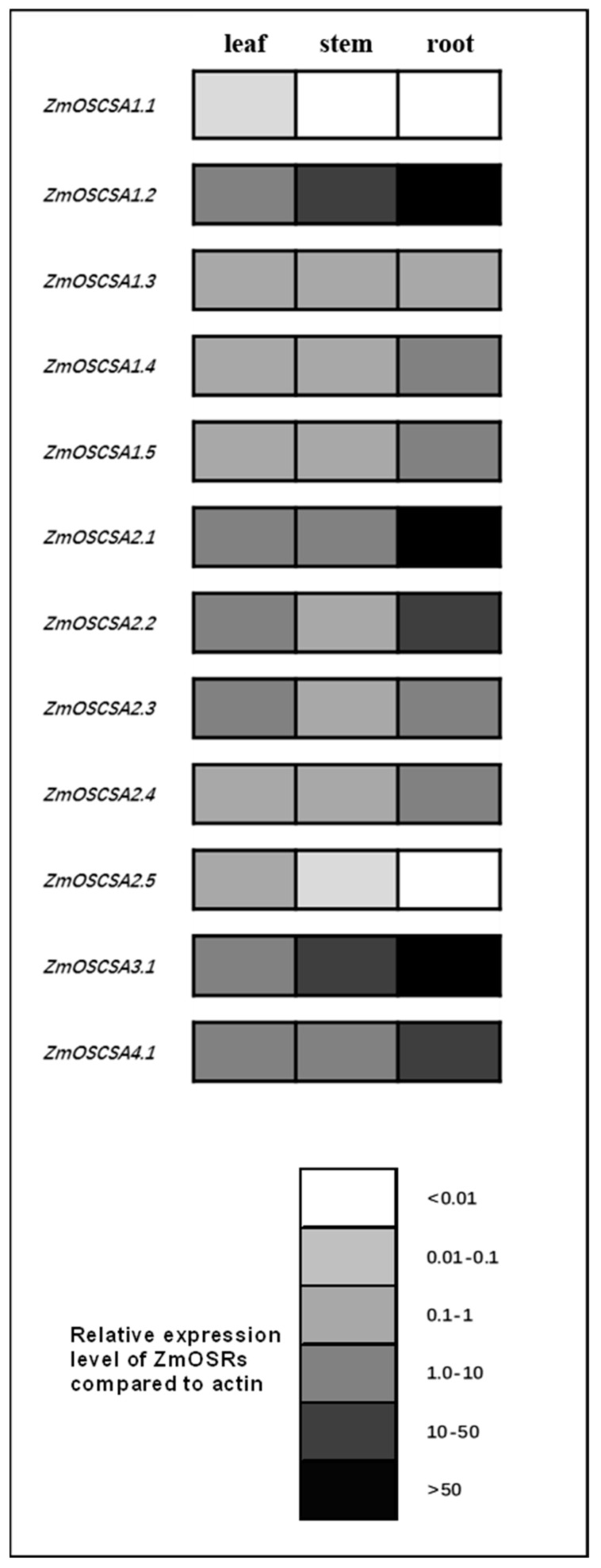
Schematic representation of the organ-specific expression of the *ZmOSCA* genes. The colors white, 25% grey, 35% grey, 50% grey, 75% grey, and black represent the multiple ranges of *ZmOSCA* mRNA expression levels, which were <0.01, 0.01–0.1, 0.1–1, 1.0–10, 10–50, and >50, respectively, compared with 0.002 *GAPDH*.

To unveil the potential function of *ZmOSCAs* in maize, the expression profiles of *ZmOSCA* genes in various tissues and organs were first determined using RT-PCR (real-time reverse transcription-PCR), and are represented in greyscale to facilitate visualization. The twelve *ZmOSCA* genes showed tissue-specific expression patterns. *ZmOSCA* genes were highly expressed in all tissues tested with two exceptions (Figure 5), which is indicative of a universal role of *OSCAs* in osmotic-sensing processes throughout the plant. *ZmOSCA2.3* and *ZmOSCA2.5* were detected only in the leaf, indicative of a specific function therein. *ZmOSCA1.3*, *ZmOSCA1.4*, *ZmOSCA1.5*, *ZmOSCA2.3*, and *ZmOSCA2.4* showed medium transcript abundances in all tissues tested. *ZmOSCA1.2*, *ZmOSCA2.1*, and *ZmOSCA3.1* had high transcript abundance in the root and relatively high transcript abundance in the stem, suggesting that they are likely related to root uptake. These results indicate that *ZmOSCAs* are involved in various physiological and developmental processes in maize.

### 2.5. Expression Profiles of ZmOSCAs under Osmotic-Related Abiotic Stresses

**Figure 6:**
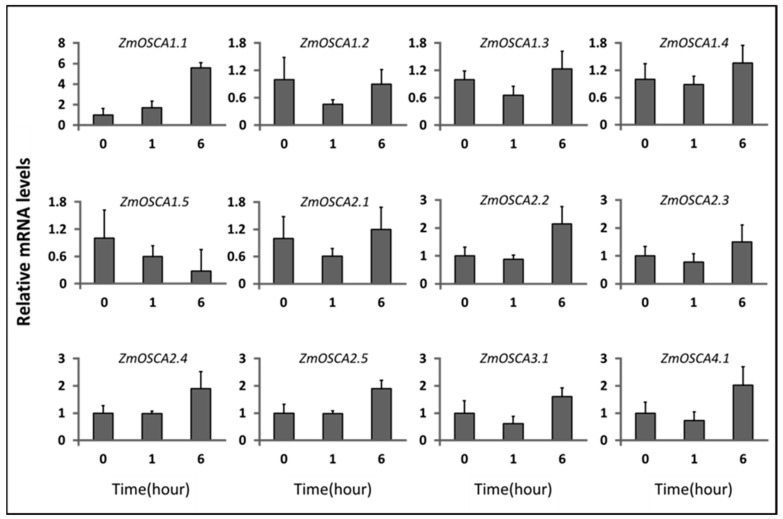
Expression profiles of *ZmOSCA* genes under 10% PEG-6000. The relative expression levels of *ZmOSCA*s were determined in the roots of three-true-leaf-stage seedlings treated with 10 % PEG-6000 for 0 h, 1 h, and 6 h and compared with the control.

**Figure 7:**
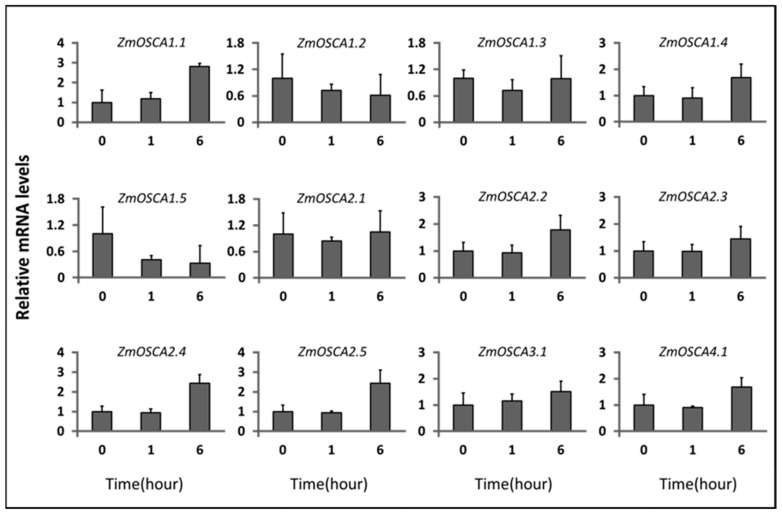
Expression profiles of *ZmOSCA* genes under NaCl stress. The relative expression levels of *ZmOSCA*s were determined in the roots of three-true-leaf-stage seedlings treated with 200 mM NaCl.

To determine whether the expression of *ZmOSCAs* is responsive to osmotic-related abiotic stress, qRT-PCR analysis of the *ZmOSCAs* at the 10-day stage in maize was performed under PEG 6000 (10%) and NaCl (250 mM). We found that twelve *ZmOSCA* genes were down- or upregulated in the stress conditions examined as compared with the control (Figure 6 and Figure 7). Majority of the *ZmOSCAs* were substantially altered within 6 h. *ZmOSCA1.1* was upregulated by 10% PEG and salt stress treatment, and *ZmOSCA1.5* was specifically downregulated. *ZmOSCA1.3* and *ZmOSCA2.1* were downregulated at 1 h and then slowly recovered. Among the genes expressed after treatment, *ZmOSCA1.1* was the most altered. These results indicate that *ZmOSCAs* might be involved in osmotic-related signaling pathways.

### 2.6. Expression Profile of ZmOSCAs under Environment-Related Abiotic Stresses

**Figure 8:**
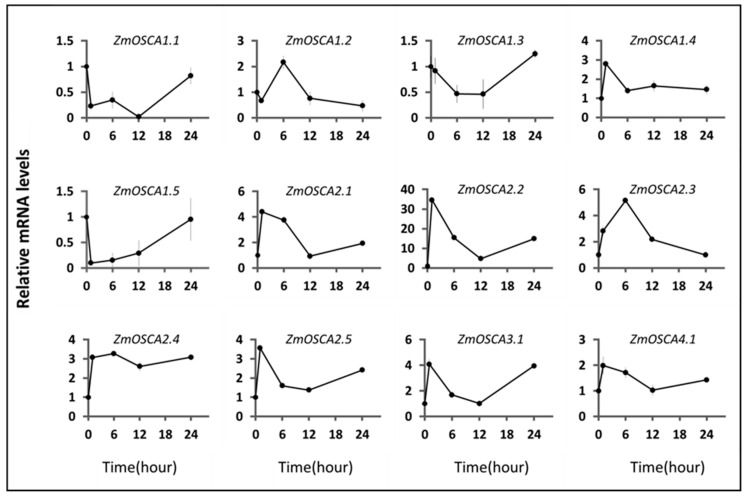
The expression levels of *ZmOSCA* genes under 40 °C treatment conditions were monitored using qRT-PCR. Samples were collected after 0, 1, 6, 12, and 24 h of heat treatment.

**Figure 9:**
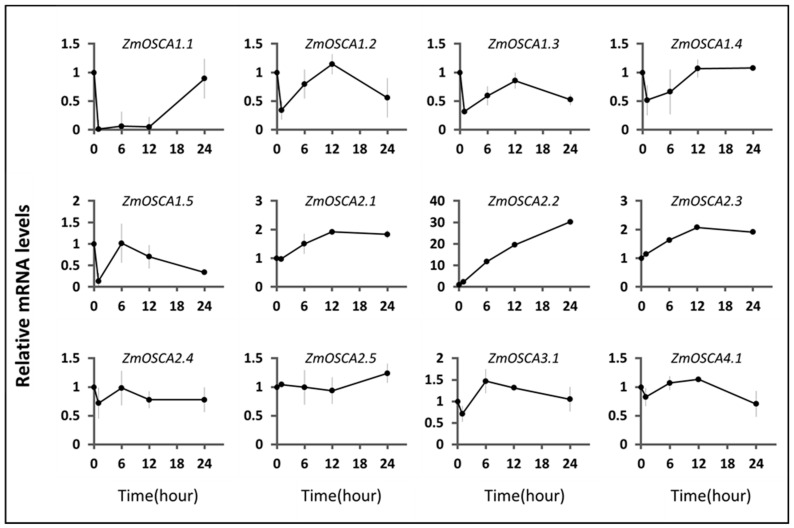
The expression levels of *ZmOSCA* genes under 4 °C treatment conditions were monitored with qRT-PCR. Samples were collected after 0, 1, 6, 12, and 24 h of cold treatment.

To further investigate whether *ZmOSCAs* affect other environmentally relevant abiotic stresses, we used qRT-PCR analysis at the 10-day stage in maize, performed at 4 °C and 40 °C. The test results showed that twelve *ZmOSCA* genes were changed in the conditions examined as compared with the control (Figure 8 and Figure 9).

When the plants were under 40 °C stress, we found that the expression of *OSCA* genes could be divided into two groups. The expression trends of six genes, *ZmOSCA1.4*, *ZmOSCA2.1*, *ZmOSCA2.2*, *ZmOSCA2.5*, *ZmOSCA3.1*, and *ZmOSCA4.1*, were relatively similar; all of them showed a significant increase in gene expression level after 1 h of high-temperature stress, reached a peak, and then were rapidly downregulated, followed by a slow increase. The expression trend of *ZmOSCA2.1* was similar to that of *ZmOSCA2.3*, except that the expression level continued to be downregulated until 24 h of treatment, and did not increase. The expression trend of *ZmOSCA1.2* was similar, with the difference that expression was slightly downregulated at 1 h of high-temperature stress and then increased continuously.

The expression patterns of *ZmOSCA1.3*, *ZmOSCA1.5*, and *ZmOSCA2.4* were opposite to the other members of the maize *OSCA* family. The expression of *ZmOSCA1.3* continued to be downregulated after high-temperature stress in maize, and started to show an upregulation trend after reaching a minimum value at 6–12 h. By 24 h, it exceeded its level in the absence of stress. *ZmOSCA1.5* expression dropped to a very low level within 1 h of heat stress, then increased slowly during the next 23 h. The initial expression level was restored after 24 h of stress. The expression image of *ZmOSCA2.4* showed that after the rapid upregulation of expression level induced by high temperature, the transcript level of this gene continued to be stable at the level above and below three times the level of the initial expression.

From the data obtained from seedlings under low-temperature stress at 4 °C, we know that for the vast majority of *ZmOSCA* genes (*ZmOSCA1.2*, *ZmOSCA1.3*, *ZmOSCA1.5*, *ZmOSCA2.4*, *ZmOSCA3.1*, and *ZmOSCA4.1*), expression decreases first, then rises to a relatively large value, then gradually decreases again. ZmOSCA1.4 is very similar to this trend, except that it rises to a value and then does not fall. Similarly, *ZmOSCA2.1* is not obvious at the beginning of the decline. Their function in plant resistance to low temperatures is similar as well. More special are *ZmOSCA1.1*, *ZmOSCA2.2*, and *ZmOSCA2.3. ZmOSCA1.1* showed a cliff-like decrease and only slowly recovered after 12 h. The expressions of *ZmOSCA2.2* and *ZmOSCA2.3*, in general, increased steadily. The expression level of *ZmOSCA3.1* at 6 h reached 1.5-fold that in the absence of stress, and we speculate that the transcription of this gene may be regulated by the feedback mechanism of low-temperature stress and involved in a specific function in the low-temperature stress response.

### 2.7. Prediction of Binding Sites for Ca^2+^ in OSCA Proteins

**Figure 10:**
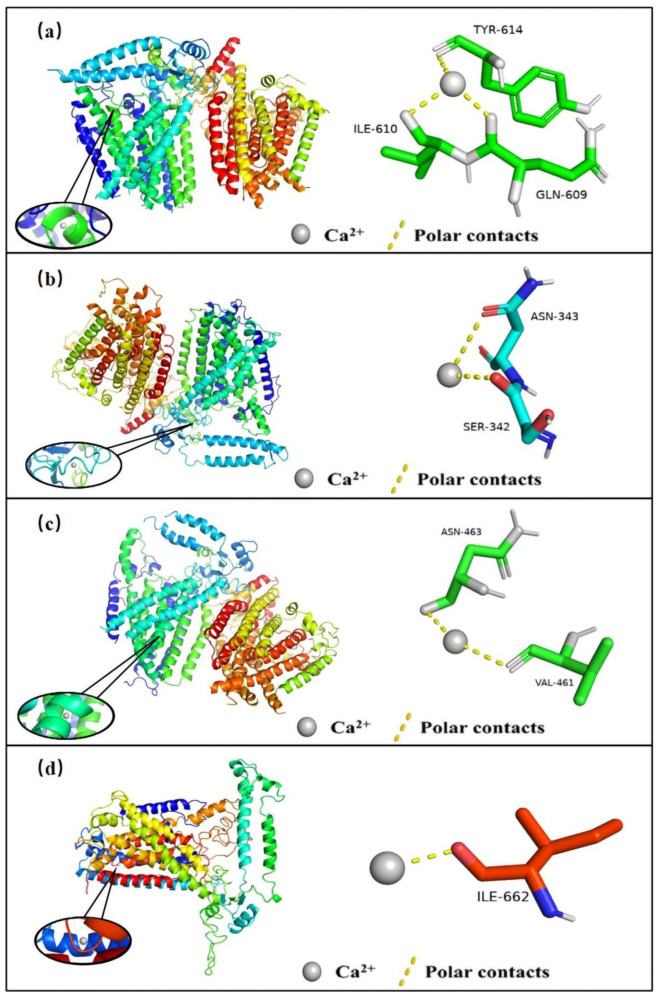
The possible binding mode of Ca^2+^ and OSCAs was simulated by Auto Dock 4.0 software. (**a**) Ca^2+^ binds to residues GLN-609, ILE-610, and TRY-614 by a covalent bond in OSCA1.1 (**b**) Ca^2+^ binds to residues SER-342 and ASN-343 by a covalent bond in OSCA2.1 (**c**) Ca^2+^ binds to residues ASN-463 and VAL-461 by a covalent bond in OSCA3.1 (**d**) Ca^2+^ bind to residues ILE-662 by a covalent bond in OSCA4.1.

As the crystal structure of ZmOSCAs has not been reported, we used the OSCA1.1 of Arabidopsis. We acquired the projected structure of this protein by SWISS-MODEL Repository (OSCA1.1, ID: 6JPF) and modeled the likely binding mode of calcium ions to the OSCA proteins using molecular docking. There are different potential binding sites between calcium ions and OSCAs through polar contacts, as shown in Figure 10. According to the results of the images, we conclude that OSCA is often combined with ASN and ILE. Such a phenomenon may be associated with the charge distribution of protein amino acid residues. Perhaps a deeper study of OSCA, a calcium-permeable protein, could be performed through calcium-binding sites.

### 2.8. ZmOSCA2.2 and ZmOSCA2.3 Protein Prediction Analysis Results

**Figure 11:**
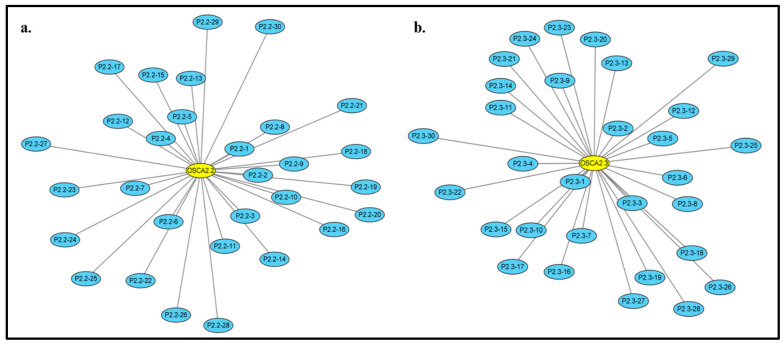
Predicted protein interaction results for ZmOSCA2.2 and ZmOSCA2.3. (**a**) Predictive analysis results of ZmOSCA2.2 and (**b**) Predictive analysis results of ZmOSCA2.3. The length of the line segment indicates the distance between the target protein and the predicted protein in terms of the action relationship. No second shell is set to interact with the protein.

Based on the *OSCA* genes affected by temperature stress, we selected ZmOSCA2.2 and ZmOSCA2.3, for which the expression was significantly altered at both 40 °C and 4 °C. These were further analyzed in order to predict protein interactions (Figure 11; Table 4 and Table 5). From the predictive analysis, we know that the PPI enrichment *p*-value is 9.36 × 10^-5^ for ZmOSCA2.2, while for ZmOSCA2.3 PPI it is 6.58 × 10^-8^. The mean clustering coefficients of ZmOSCA2.2 and ZmOSCA2.3 are 0.793 and 0.855, respectively.

Looking at the predicted results, our analysis perceives that both ZmOSCA2.2 and ZmOSCA2.3 have interactions with GRMZM2G029546, GRMZM2G045854, GRMZM2G033926, GRMZM2G175423, GRMZM2G702253, GRMZM2G060296, and GRMZM2G319454. Analysis of its protein annotation and family revealed that GRMZM2G029546 is a Dnaj heat shock n-terminal domain-containing and Electron transporter/heat shock protein binding protein with important roles in plant stress responses and signaling. GRMZM2G045854 is a Gbf-interacting protein, which has a DUF1296 domain, which can enhance the transcriptional activity of plant growth-related transcription factors [22]. GRMZM2G033926 belongs to the 1-acyl-sn-glycerol-3-phosphate acyltransferases, which can catalyze the acylation of lysophosphatidic acid and are responsible for the de novo production of phosphatidic acid [23]. GRMZM2G175423 belongs to the class of Sorbitol dehydrogenases. For enzymes, including many proteins that are irreplaceable in biological reactions, the differences they show with changes in temperature are extremely significant. OSCA has several interactions with a wide range of enzymes in maize.

Moreover, OSCA2.2 interacts closely with the NAD(P)-dependent dehydrogenase, short-chain alcohol dehydrogenase family and allantoin: proton symporter activity. As such, we speculate that OSCA2.2 is related to energy metabolism. In the prediction results of OSCA2.3, two protein families, pectinesterase inhibitor activity and ethylene-activated signaling pathway, were prominent. We speculate that OSCA2.3 is involved in the hormone pathway in plants and may play a specific role. Furthermore, OSCA2.3 was found to be associated with ABC transporter protein. In Gram-Positive Pathogens, the ABC protein is governed by the treatment temperature [24].

In addition, botanists have shown that the protein expression of ABC transporter protein changes in plant leaves with the impact of temperature [25]. These findings offer additional proof that OSCA is involved in many physiological activities in plants.

Finally, OSCA might be temperature-related in a way that we do not yet know. Our work demonstrates the dependence between OSCA and temperature, and provides evidence to further investigate the temperature mechanism of plants.

## 3. Discussion

Ion channels are essential for cell survival, and cells use them for nutrient uptake, sensing environmental stimuli, and signaling [26]. Many different stimulus-sensing proteins exist in plants to sense different stress signals; the functional proteins of the early signaling phase of plant stress signal transduction are unknown. However, it has been demonstrated that low temperature, drought, and high salt stress can induce transient calcium inward flow in the cytoplasm. Thus, it can be inferred that the ion channels causing inward calcium flow are likely to act as a class of receptors for stress signals [27]. The Sodium Proton Antiporter (NHX) temperature of Arabidopsis has previously been found to affect the translation of its gene family, while predictive analysis found that SbNHX is closely linked to the calcineurin B-like proteins (CBL)-interacting protein kinases (CIPK) pathway [28]. This further suggests that calcium proteins may be temperature-dependent. Calcium transporter protein ANN1 was found to be involved in plant cold-induced responses in *Arabidopsis* [29], suggesting that calcium transporter proteins can be directly involved in plant stress responses due to temperature changes. We found differences in the expression levels of the twelve *ZmOSCA* genes under high and low temperature conditions, something that has never previously been suggested.

In addition to the above, there are many transcription factors associated with osmoregulation in plants, such as DREB, MYC/MYB, and WRKY [30]. As osmoregulation-related transcription factors, they have been found to play a role in osmosis and to be inextricably linked to other metabolic pathways in plants. DREB2A regulates plant heat stress response by regulating the cascade response of the heat stress transcription factor HsfA [31]. MYB/MYC, the largest family of transcription factors in plants [32], can alter the regulation of stomatal movement and is involved in physiological regulation related to plant sensibility [33]. WRKY has been shown to have an important relationship with the response to high-temperature stress. WRKY factors can bind to the W-box cis-acting elements of target gene promoters, thereby regulating the expression of multiple target genes and participating in multiple signaling pathways in plants [34]. While *OSCAs* are found to be associated with osmosis, they should not act only in osmoregulation, as with the factors mentioned above. Three *OsOSCAs* are associated with circadian regulation, and are related to transpiration regulation [20]. The *SlOSCA* gene regulates light responses and contains elements involved in the light pathway [35]. Based on these results, the role of *OSCA*s stretches far beyond what has been found to date. *OSCA*s encode genes that may play a decisive role in other processes of plant growth and development, and these questions require further investigation. In the case of heat stress, changes in other factors provide us with a new viewpoint about *OSCA*s; based on existing knowledge of *OSCA*s, we assume that they have a high probability of reacting to thermal stress.

The evolutionary tree we constructed based on the proteins tells us that *OSCA*s can be classified into four categories, which is consistent with the evolutionary analysis of Arabidopsis, rice, poplar, and grapevine [16]. The *OSCA* gene family of the typical monocot plant rice and the dicotyledonous plant Arabidopsis is present in each category, showing that the *OSCA* gene family has the potential to diversify before plant differentiation. We found that the second subfamily of *ZmOSCA* was expressed in a similar trend in cold stress, which is similar to the results of the promoter analysis in [17]. Thus, we believe that OSCA subfamily II may be a group with less alteration in the biological evolution of the OSCA gene family in terms of their protein reaction to temperature stress. This may play a decisive role in the investigation of species genesis.

Through predictive analysis of *Zm*OSCA protein interactions, we found that *Zm*OSCA2.3 is closely associated with catabolic processes such as amino acids, cellular nitrogen compounds, and organic cyclic compounds, suggesting that *Zm*OSCA2.3 is involved in many metabolic activities. This echoes the OSCA2.3 related study of Thor. et al. [36]. These data strengthen the credibility of our protein interactions prediction analysis. At the same time, we discovered that *Zm*OSCA involves enzymatic reactions in a variety of organisms. The temperature–enzyme relationship gives us more confidence that *Zm*OSCA is a protein involved in temperature regulation. In fact, several in vivo transporters have a co-operative relationship with *Zm*OSCA2.2, and many of these are transmembrane-associated protein transporters. The effect of temperature on the association between plant plasma membrane transport and protein transporters has been well documented in plant physiology. In addition, the properties of protein denaturation at high temperatures allows us to be more certain that *Zm*OSCA is highly temperature modulation-related.

At present, we have not explored the specific targets of the *OSCA* signaling pathway under temperature stress via other experiments, and this is the direction we are considering following in continuing our in-depth study in the future. In our protein interactions prediction, we found that *OSCA* is closely linked to other metabolic pathways, and in the future we will consider validating the role of *OSCA* within other metabolic pathways in order to explore and discuss more functions that *OSCA* may have. This protein could be interchangeable with yield-related influences in experiments to help solve the problem of world hunger.

## 4. Materials and Methods

### 4.1. Bioinformatics Analysis

#### 4.1.1. Gene Screening and Identification Analysis

Identified *Arabidopsis OSCA* gene family gene IDs were downloaded from the National Center for Biotechnology Information (https://www.ncbi.nlm.nih.gov/, revised 17 January 2022), and candidate genes were identified by tBlast N searches in their maize genome library. The *ZmOSCA* gene family members were identified using the Pfam and SMART databases and validated by the DUF221 structural domain. When multiple transcripts existed at the same gene locus, the deduced amino acid sequence of the longest transcript was selected for comparison. Prediction of genomic exons and introns was carried out using Gene Structure Display Server (GSDS, http://gsds.cbi.pku.edu.cn/, revised 26 January 2022).

#### 4.1.2. Construction of Evolutionary Tree and Analysis of Gene Structure

The full-length proteins were analyzed using MEGA5.0 software, and phylogenetic trees were constructed by the Neighbor Joining (NJ) method [37]. A total of 5000 bootstrap replicates were performed in each analysis to obtain confidential support. Gene structures of *OSCAs* were identified using the Gene Structure Display Server. Multiple sequence alignments of *OSCAs* were performed using DNAMAN, and the transmembrane region of the DUF221 conserved region was predicted using TMHMM.

#### 4.1.3. Analysis of Protein Secondary Structure Prediction in Maize *OSCA* Family

Based on the principle of homology prediction, the protein secondary structure prediction website SOPMA (https://npsa-prabi.ibcp.fr/cgi-bin/npsa_automat.pl?page=/NPSA/npsa_sopma.html, revised 2 March 2022) was used to predict the secondary structure of *OSCA* in maize based on the relevant protein database. The secondary structure of each member of the *OSCA* protein family was predicted based on the relevant protein database, and the results were checked statistically. The TM domains in *OSCAs* were annotated according to TMHMM Server v. 2.0 predictions (http://www.cbs.dtu.dk/services/TMHMM/, revised 8 March 2022).

#### 4.1.4. Predictive Analysis of Protein Interactions

Using the STRING website (https://string-db.org/, revised 18 March 2022), the minimum required interaction score was manually set to 0.4 and the first interacting protein shell parameter was set to 30 to construct the protein interaction prediction analysis data, then edited with Cytoscape software and checked with protein information from the National Center for Biotechnology Information (https://www.ncbi.nlm.nih.gov/, revised 18 March 2022).

#### 4.1.5. Prediction of Recognition Sites for Calcium Ions

The OSCA protein structure was located and downloaded from the PDB database (https://www.rcsb.org/, accessed on 22 March 2022); using the SWISS-MODEL website (https://swissmodel.expasy.org/, accessed 24 March 2022), the remaining several OSCA structures that were not yet measured were constructed homologously using AtOSCA1.1 (ID: 6JPF) as a template [38]. The proteins were then processed using Auto Dock 4.0 [39] for mapping and analysis based on the system-defined highly probable docking sites.

### 4.2. Plant Materials and Growth Conditions

Maize plants (B73) were planted in a growth chamber at Jilin University (Changchun, China). Plants were surface sterilized in a 10% (*v*/*v*) H_2_O_2_ solution for 20 min and germinated on sand for 3 days in a growth chamber at 4 °C with a 10 h/14 h light/dark cycle and 70–80% relative humidity. The seeds were transferred into improved Hoagland solution at 28/25 °C (day/night) with a photoperiod of 16/8 h (day/night) and relative humidity of 50–70%; the nutrient solution was replaced regularly with a new one every day. When10 days old, maize seedlings were placed in a nutrient solution containing 10% PEG for drought stress treatment; seedlings were treated with a low temperature of 4 °C and a high temperature of 40 °C for 24 h in the light incubator and taken at 0 h, 1 h, 6 h, 12 h, and 24 h after treatment. Maize seedlings were treated with 250 mmol/L NaCl solution for salt stress, and the seedlings were taken at 0 h, 1 h, and 6 h, after treatment. The nutrient solution was renewed daily.

### 4.3. RNA Extraction and Quantitative qRT-PCR Analysis

Extraction of maize RNA was carried out by the TRIzol method. Reverse transcription kits from Invitrogen were then used to synthesize the first strand cDNA. cDNA template was diluted and used and real-time fluorescence quantitative PCR was performed using the SYBR GREEN method as provided by the company’s QRT kit. Finally, the relative amount of gene expression was calculated using the 2^−ΔΔCt^ method [40]. For detailed primer sequences, see Table S1

## 5. Conclusions

We found evidence for the involvement of *ZmOSCAs* in temperature correlation, along with an exhaustive analysis of the *ZmOSCA* family. We found that the *OSCA* gene family of maize is characterized by tissue-specific expression, which is highly consistent with studies of *OSCA* in rice [20]. This is the first discovery in maize, and represents a further exploration of the molecular mechanisms underlying the interactions of *ZmOSCA* under adversity stresses. Further investigation of the regulatory mechanism of ZmOSCA2.2 and ZmOSCA2.3 in the cold/heat stress response can contribute to the engineering of cold/heat-tolerant crops via element-specific genome editing.

## Figures and Tables

**Table 1:** Information of *OSCA* family members in maize.

Gene	Position	Number of Transcripts	Number of Exons	Length (AA)	Strand
*ZmOSCA1.1*	Contig B73V4_ctg31 243,552–267,664	9	11	768	forward strand
*ZmOSCA1.2*	Chr3 231,673,936–231,685,374	15	13	785	forward strand
*ZmOSCA1.3*	Chr8 80,433,878–80,440,706	6	12	790	forward strand
*ZmOSCA1.4*	Chr6 152,198,261–152,212,313	14	12	748	reverse strand
*ZmOSCA1.5*	Chr1 108,282,883–108,288,994	5	11	810	reverse strand
*ZmOSCA2.1*	Chr3 106,709,675–106,715,943	6	12	767	forward strand
*ZmOSCA2.2*	Chr1 261,919,725–261,930,103	2	12	765	forward strand
*ZmOSCA2.3*	Chr5 9,735,399–9,740,575	8	11	749	forward strand
*ZmOSCA2.4*	Chr1 164,016,950–164,031,018	5	10	699	forward strand
*ZmOSCA2.5*	Chr8 163,438,578–163,443,056	3	10	703	reverse strand
*ZmOSCA3.1*	Chr2 241,256,310–241,261,093	1	6	732	reverse strand
*ZmOSCA4.1*	Chr9 155,107,951–155,110,341	1	1	796	forward strand

**Table 2:** The distribution of plant *OSCA* genes in groups Ⅰ, Ⅱ, Ⅲ, and Ⅳ.

Species	Total	Group Ⅰ	Group Ⅱ	Group Ⅲ	Group Ⅳ
*Arabidopsis thaliana*	15	8	5	1	1
*Oryza sativa*	11	4	5	1	1
*Zea mays*	12	5	5	1	1

**Table 3:** Predicted secondary structure of maize OSCA proteins.

Gene	Alpha Helix (Hh)		Extended Strand (Ee)		Beta Ture (Tt)		Random Coil (Cc)	
*ZmOSCA1.1*	317	41.28%	178	23.18%	44	5.73%	229	29.82%
*ZmOSCA1.2*	331	42.17%	175	22.29%	46	5.86%	233	29.68%
*ZmOSCA1.3*	338	42.78%	170	21.52%	51	6.46%	231	29.24%
*ZmOSCA1.4*	302	40.37%	167	22.33%	60	8.02%	219	29.28%
*ZmOSCA1.5*	380	46.91%	151	18.64%	50	6.17%	229	28.27%
*ZmOSCA2.1*	322	41.98%	173	22.56%	50	6.52%	222	28.94%
*ZmOSCA2.2*	292	28.17%	166	21.70%	53	6.93%	254	33.20%
*ZmOSCA2.3*	337	44.99%	165	22.03%	63	8.41%	184	24.57%
*ZmOSCA2.4*	315	45.06%	147	21.03%	42	6.01%	195	27.90%
*ZmOSCA2.5*	332	47.23%	128	18.21%	56	7.97%	187	26.60%
*ZmOSCA3.1*	329	44.95%	154	21.04%	52	7.10%	197	26.91%
*ZmOSCA4.1*	400	50.25%	122	15.33%	50	6.28%	224	28.14%

**Table 4:** Prediction of proteins interacting with OSCA2.2 and their family information.

	Identifier	Protein Annotations	Length (AA)	Protein Families (COGs):	Protein Families Function
P2.2-1	GRMZM2G072052_P01	Putative calcium-binding protein CML15	234	COG5126	Ca^2+^ -binding protein, EF-hand superfamily
P2.2-2	GRMZM2G121034_P01	NAD(P)-binding Rossmann-fold superfamily protein; Retinol dehydrogenase 12; Uncharacterized protein; Belongs to the short-chain dehydrogenases/reductases (SDR) family	367	COG1028	NAD(P)-dependent dehydrogenase, short-chain alcohol dehydrogenase family
P2.2-3	GRMZM2G450125_P01	Beta-amylase	573	NOG256053	Amylopectin maltohydrolase activity
P2.2-4	GRMZM2G177570_P01	Annotation not available	707	COG4886	Leucine-rich repeat (LRR) protein
P2.2-5	GRMZM2G046111_P01	Plant invertase/pectin Methylesterase inhibitor superfamily protein	243	NOG10079	Enzyme inhibitor activity
P2.2-6	GRMZM2G045854_P01	Gbf-interacting protein 1 isoform x1; Putative DUF1296 domain-containing family protein	540	NOG236453	Protein of unknown function (DUF1296)
P2.2-7	GRMZM2G033926_P01	WAPL (Wings apart-like protein Regulation of heterochromatin) protein	334	COG0204	1-acyl-sn-glycerol-3-phosphate acyltransferase
P2.2-8	GRMZM2G180054_P01	Putative calcium-binding protein CML15	109	COG5126	Ca^2+^-binding protein, EF-hand superfamily
P2.2-9	GRMZM2G025833_P01	Beta-amylase	544	NOG256053	Amylopectin maltohydrolase activity
P2.2-10	GRMZM5G847466_P01	Calcium-binding protein CML; Calmodulin	172	COG5126	Ca^2+^-binding protein, EF-hand superfamily
P2.2-11	GRMZM2G147014_P01	Dehydrin COR410; Belongs to the plant dehydrin family	290	NOG17228	Response to water
P2.2-12	GRMZM2G474777_P01	Tyrosine-sulfated glycopeptide receptor 1; Putative phytosulfokine receptor (LRR repeat-containing protein kinase) family protein	665	COG4886	Leucine-rich repeat (LRR) protein
P2.2-13	GRMZM2G154114_P01	Annotation not available	381	COG0515	Serine/threonine-protein kinase
P2.2-14	GRMZM2G373522_P01	Dehydrin3; Dehydrin; Uncharacterized protein; Belongs to the plant dehydrin family	289	NOG17228	Response to water
P2.2-15	GRMZM2G095452_P01	Transcription repressor OFP12	269	NOG269813	Negative regulation of transcription, DNA-templated
P2.2-16	GRMZM2G464885_P01	Uncharacterized protein loc100192507; Short-chain dehydrogenase TIC 32 chloroplastic	314	COG1028	NAD(P)-dependent dehydrogenase, short-chain alcohol dehydrogenase family
P2.2-17	GRMZM2G043191_P01	Uncharacterized protein loc100279724; Type IV inositol polyphosphate 5-phosphatase 11	347	COG5411	Phosphatidylinositol dephosphorylation
P2.2-18	GRMZM2G175423_P01	Sorbitol dehydrogenase homolog 1; Sorbitol dehydrogenase	366	COG1063	Threonine dehydrogenase or related Zn-dependent dehydrogenase
P2.2-19	GRMZM2G009940_P02	Short-chain dehydrogenase TIC 32 chloroplastic; Retinol dehydrogenase 14; Uncharacterized protein; Belongs to the short-chain dehydrogenases/reductases (SDR) family	316	COG1028	NAD(P)-dependent dehydrogenase, short-chain alcohol dehydrogenase family
P2.2-20	GRMZM2G128577_P02	Short-chain dehydrogenase TIC 32 chloroplastic; Belongs to the short-chain dehydrogenases/reductases (SDR) family	315	COG1028	NAD(P)-dependent dehydrogenase, short-chain alcohol dehydrogenase family
P2.2-21	GRMZM2G702253_P02	Annotation not available	304	COG0345	Pyrroline-5-carboxylate reductase
P2.2-22	GRMZM2G119079_P01	Dual-specificity protein-like phosphatase 1; Protein-tyrosine phosphatase mitochondrial 1	347	KOG1719	Dual specificity phosphatase
P2.2-23	GRMZM2G029546_P03	Dnaj heat shock n-terminal domain-containing protein; Electron transporter/heat shock protein-binding protein; Uncharacterized protein	343	COG0484	DnaJ-class molecular chaperone with C-terminal Zn finger domain
P2.2-24	GRMZM2G055238_P01	Uncharacterized loc100272659; Uncharacterized protein	403	NOG06404	Allantoin: proton symporter activity
P2.2-25	GRMZM2G135746_P01	Uncharacterized protein; Ureide permease 5	403	NOG06404	Allantoin: proton symporter activity
P2.2-26	GRMZM2G060296_P01	Signal recognition particle receptor subunit alpha; Signal recognition particle protein subunit 9	625	COG0552	Signal recognition particle GTPase
P2.2-27	GRMZM2G025528_P05	Uncharacterized protein; Ureide permease 5	242	NOG06404	Allantoin: proton symporter activity
P2.2-28	GRMZM2G091891_P02	Uncharacterized loc100502471; Ureide permease 5	411	NOG06404	Allantoin: proton symporter activity
P2.2-29	GRMZM2G022837_P01	Menaquinone-specific isochorismate synthase; Isochorismate synthase 2 chloroplastic	562	COG0147	Anthranilate/para-aminobenzoate synthases component I
P2.2-30	GRMZM2G319454_P01IDP592	Uncharacterized protein loc100278112; Transmembrane-like protein	170	KOG4831	Unnamed protein

**Table 5:** Prediction of proteins interacting with OSCA2.3 and their family information.

	Identifier	Protein Annotations	Length (AA)	Protein Families (COGs):	Protein Families Function
P2.3-1	GRMZM6G732597_P01	Annotation not available	732	COG5594	Calcium-activated cation channel activity
P2.3-2	AC186577.3_FGP006	Pectinesterase inhibitor 28; Cell wall/vacuolar inhibitor of fructosidase 2	-	NOG14257	Pectinesterase inhibitor activity
P2.3-3	GRMZM2G368698_P02	Cell wall/vacuolar inhibitor of fructosidase 2; C/VIF2; Uncharacterized protein	214	NOG14257	Pectinesterase inhibitor activity
P2.3-4	GRMZM2G300141_P01	Cell wall/vacuolar inhibitor of fructosidase 2; Uncharacterized protein	222	NOG14257	Pectinesterase inhibitor activity
P2.3-5	GRMZM2G101945_P01	Pectinesterase inhibitor 28; Cell wall/vacuolar inhibitor of fructosidase 2	233	NOG14257	Pectinesterase inhibitor activity
P2.3-6	GRMZM5G891247_P01	Uncharacterized protein; Cell wall/vacuolar inhibitor of fructosidase 2	222	NOG14257	Pectinesterase inhibitor activity
P2.3-7	GRMZM2G055180_P01	Ethylene-responsive transcription factor 2	279	NOG243370	Ethylene-activated signaling pathway
P2.3-8	GRMZM2G466044_P01	Annotation not available	282	NOG243370	Ethylene-activated signaling pathway
P2.3-9	GRMZM2G033926_P01	WAPL (Wings apart-like protein regulation of heterochromatin) protein	334	COG0204	1-acyl-sn-glycerol-3-phosphate acyltransferase
P2.3-10	GRMZM2G358139_P01	Annotation not available	282	NOG01264	S-adenosylmethionine-dependent methyltransferase activity
P2.3-11	GRMZM2G059801_P01	Annotation not available	325	COG0484	DnaJ-class molecular chaperone with C-terminal Zn finger domain
P2.3-12	GRMZM2G080516_P01	AP2-EREBP transcription factor; Putative AP2/EREBP transcription factor superfamily protein; Uncharacterized protein	270	NOG243370	Ethylene-activated signaling pathway
P2.3-13	GRMZM2G029546_P03	Dnaj heat shock n-terminal domain-containing protein; Electron transporter/heat shock protein-binding protein; Uncharacterized protein	343	COG0484	DnaJ-class molecular chaperone with C-terminal Zn finger domain
P2.3-14	GRMZM2G343149_P01	DNAJ heat shock N-terminal domain-containing protein; DnaJ domain-containing protein; Uncharacterized protein	473	COG0484	DnaJ-class molecular chaperone with C-terminal Zn finger domain
P2.3-15	AC204277.3_FGP006	Uncharacterized loc100383917; Putative methyltransferase PMT23	-	NOG01264	S-adenosylmethionine-dependent methyltransferase activity
P2.3-16	GRMZM2G045249_P01	Probable methyltransferase pmt23 isoform x1; Putative methyltransferase PMT23	508	NOG01264	S-adenosylmethionine-dependent methyltransferase activity
P2.3-17	GRMZM2G473960_P01	Uncharacterized protein loc100286326;Seed maturation protein	112	NOG44451	Late embryogenesis abundant (LEA) group 1
P2.3-18	GRMZM2G060296_P01	Signal recognition particle receptor subunit alpha; Signal recognition particle protein subunit 9	625	COG0552	Signal recognition particle GTPase
P2.3-19	GRMZM2G054465_P01	Uncharacterized protein loc109623449; Tryptophan synthase beta type 2	409	COG0133	Tryptophan synthase beta chain
P2.3-20	GRMZM2G087758_P01	Chaperone DnaJ-domain superfamily protein; DnaJ domain-containing protein; Uncharacterized protein	258	COG0484	DnaJ-class molecular chaperone with C-terminal Zn finger domain
P2.3-21	GRMZM2G064898_P01	ORMDL family protein	162	COG5081	Negative regulation of ceramide biosynthetic process
P2.3-22	GRMZM2G319454_P01	Uncharacterized protein loc100278112; Transmembrane-like protein	170	KOG4831	Unnamed protein
P2.3-23	GRMZM2G044720_P01	Uncharacterized loc100273730; ORMDL family protein	162	COG5081	Negative regulation of ceramide biosynthetic process
P2.3-24	GRMZM2G125072_P01	DNAJ heat shock N-terminal domain-containing protein; 3Fe-4S ferredoxin; Uncharacterized protein	304	COG0484	DnaJ-class molecular chaperone with C-terminal Zn finger domain
P2.3-25	GRMZM2G045854_P01	Gbf-interacting protein 1 isoform x1; Putative DUF1296 domain-containing family protein	540	NOG236453	Protein of unknown function (DUF1296)
P2.3-26	GRMZM2G478664_P01	Annotation not available	1058	COG0060	Isoleucyl-tRNA synthetase
P2.3-27	GRMZM2G175423_P01	Sorbitol dehydrogenase homolog 1; Sorbitol dehydrogenase	366	COG1063	Threonine dehydrogenase or related Zn-dependent dehydrogenase
P2.3-28	GRMZM2G702253_P02	Annotation not available	304	COG0345	Pyrroline-5-carboxylate reductase
P2.3-29	GRMZM2G474326_P01	Pathogenesis-related genes transcriptional activator pti5; Ethylene-responsive transcription factor 2	229	NOG243370	Ethylene-activated signaling pathway
P2.3-30	GRMZM5G820122_P01	Abc transporter c family mrp4 precursor; ABC transporter that may affect phytic acid transport and compartmentalization.	1510	COG1132	The ABC-type multidrug transport system, ATPase and permease component

## Data Availability

The original contributions presented in this study are included in the article.

## Supplementary Materials

The following supporting information can be downloaded at: https://www.mdpi.com/article/10.3390/ijms232113658/s1.
