# Supplementary material for: Preliminary Expression Analysis of the OSCA Gene Family in Maize and Their Involvement in Temperature Stress
Source: Int J Mol Sci. 2022 Nov 7;23(21):13658. doi: 10.3390/ijms232113658 (PMC9656168; doi:10.3390/ijms232113658)
Supplement: Supplementary file 1 [file ijms-23-13658-s001.zip › ijms-1970447-supplementary.pdf]

Table S1. Sequences of oligonucleotide primers for qRT-PCR. F: forward; R: reverse.

Oligos	Primer Sequence(5'-3')
GADPH-F	ATGGCAAGCTCACTGGTATG
GADPH-R	CCAAGGAAGTCGGTAGAAAC
Zm00001d000077-F(ZmOSCA1.1-F)	TTCTTGGTTGTTTTGGTA
Zm00001d000077-R(ZmOSCA1.1-R)	AACTCACTTCACTTTCTAT
Zm00001d044555-F*(ZmOSCA1.2-F)*	TGTCTCCCTTACAGTTCG
Zm00001d044555-R*(ZmOSCA1.2-R)*	TGCCTTCTCAATCCCT
Zm00001d009771-F*(ZmOSCA1.3-F)*	AGCCAGGCAAGTGAC
Zm00001d009771-R*(ZmOSCA1.3-R)*	CTGTGGAATGGAGGG
Zm00001d038240-F*(ZmOSCA1.4-F)*	TACATCAGTCCGCAAATAA
Zm00001d038240-R*(ZmOSCA1.4-R)*	TACCAGACCAGCCATCA
Zm00001d030133-F*(ZmOSCA1.5-F)*	TCGCTGGAAAGGAG
Zm00001d030133-R*(ZmOSCA1.5-R)*	TAGGAGGCTGGTGG
Zm00001d041229-F*(ZmOSCA2.1-F)*	TGTAGATGTGCCCTTGGATA
Zm00001d041229-R*(ZmOSCA2.1-R)*	TCTTTAACACTGGACTGACCC
Zm00001d033401-F*(ZmOSCA2.2-F)*	GAGATTTGTCCCTTCCC
Zm00001d033401-R*(ZmOSCA2.2-R)*	CATCCAACCCAGCAGT
Zm00001d013364-F*(ZmOSCA2.3-F)*	CAGAGGCAGGACGATTAC
Zm00001d013364-R*(ZmOSCA2.3-R)*	GAGTCCAAGCCAGCAAC
Zm00001d030860-F*(ZmOSCA2.4-F)*	GTGAACTTCCTCGGTGAC
Zm00001d030860-R*(ZmOSCA2.4-R)*	CGTTGGAGATGCTGAATAGA
Zm00001d011872-F*(ZmOSCA2.5-F)*	GGGGCTTACGGGTGTTA
Zm00001d011872-R*(ZmOSCA2.5-R)*	ATGCCTCAATGTTTCCTATGT
Zm00001d007865-F*(ZmOSCA3.1-F)*	TGGGCATTTCTGTTGTCA
Zm00001d007865-R*(ZmOSCA3.1-R)*	ACCGCAAACTCCTCTGG
Zm00001d048361-F*(ZmOSCA4.1-F)*	CAAACAAGGCGGTAAAGG
Zm00001d048361-R*(ZmOSCA4.1-R)*	TGATTGCCAAGGGTGAACT
